# Adenoviral vectored vaccination protects against Crimean-Congo Haemorrhagic Fever disease in a lethal challenge model

**DOI:** 10.1016/j.ebiom.2023.104523

**Published:** 2023-03-17

**Authors:** Jack E. Saunders, Ciaran Gilbride, Stuart Dowall, Susan Morris, Marta Ulaszewska, Alexandra J. Spencer, Emma Rayner, Victoria A. Graham, Emma Kennedy, Kelly Thomas, Roger Hewson, Sarah C. Gilbert, Sandra Belij-Rammerstorfer, Teresa Lambe

**Affiliations:** a The Jenner Institute, Nuffield Department of Medicine, University of Oxford, Oxford, UK; b Oxford Vaccine Group, Department of Paediatrics, University of Oxford, Oxford, UK; c UK Health Security Agency (UKHSA), Porton Down, Salisbury, Wiltshire, UK; d Chinese Academy of Medical Science (CAMS) Oxford Institute, University of Oxford, Oxford, UK

**Keywords:** Vaccine, CCHFV, Heterologous immunisation, ChAdOx2, Antibodies, T cells, Prime and boost

## Abstract

**Background:**

The tick-borne bunyavirus, Crimean-Congo Haemorrhagic Fever virus (CCHFV), can cause severe febrile illness in humans and has a wide geographic range that continues to expand due to tick migration. Currently, there are no licensed vaccines against CCHFV for widespread usage.

**Methods:**

In this study, we describe the preclinical assessment of a chimpanzee adenoviral vectored vaccine (ChAdOx2 CCHF) which encodes the glycoprotein precursor (GPC) from CCHFV.

**Findings:**

We demonstrate here that vaccination with ChAdOx2 CCHF induces both a humoral and cellular immune response in mice and 100% protection in a lethal CCHF challenge model. Delivery of the adenoviral vaccine in a heterologous vaccine regimen with a Modified Vaccinia Ankara vaccine (MVA CCHF) induces the highest levels of CCHFV-specific cell-mediated and antibody responses in mice. Histopathological examination and viral load analysis of the tissues of ChAdOx2 CCHF immunised mice reveals an absence of both microscopic changes and viral antigen associated with CCHF infection, further demonstrating protection against disease.

**Interpretation:**

There is the continued need for an effective vaccine against CCHFV to protect humans from lethal haemorrhagic disease. Our findings support further development of the ChAd platform expressing the CCHFV GPC to seek an effective vaccine against CCHFV.

**Funding:**

This research was supported by funding from the 10.13039/501100000268Biotechnology and Biological Sciences Research Council (UKRI-BBSRC) [BB/R019991/1 and BB/T008784/1].

**Research in context**

**Evidence before this study**

Crimean-Congo Haemorrhagic Fever Virus (CCHFV) is a tick-borne pathogen that can cause lethal haemorrhagic disease in humans. Of particular concern is the wide geographic range of CCHFV that continues to expand due to tick migration. There are currently no widely available treatments or vaccines against CCHFV-mediated disease. A number of CCHFV vaccine candidates have been developed but progression towards clinical development has been limited for promising experimental vaccines.

**Added value of this study**

We have developed a CCHFV vaccine using chimpanzee adenovirus viral vector technology that is replication-deficient and a similar construct to that used to generate the Oxford/AstraZeneca COVID-19 vaccine (ChAdOx1 nCoV-19/AZD1222). The generated vaccine, ChAdOx2 CCHF, induces a strong immune response against the glycoprotein of CCHFV and mediates complete protection from CCHFV-mediated disease in a mouse model. We present a detailed description of the immune response after vaccination and we demonstrate that a two-dose regimen with an alternate replication-deficient viral vector, Modified Vaccinia Ankara (MVA) CCHF vaccine, induces the strongest immune response. Histopathological examination and viral load analysis provided further evidence that ChAdOx2 CCHF induced protection in vaccinated animals by the absence of detectable CCHFV and no overt disease as a result of CCHFV challenge.

**Implications of all the available evidence**

There remains the necessity for scalable, affordable and effective vaccines against CCHFV to protect susceptible individuals in endemic regions. Together, the data here support the continued preclinical and clinical development of a ChAd CCHFV vaccine as a potential candidate to produce an immunogenic and protective vaccine against CCHFV for humans.

## Introduction

Crimean-Congo Haemorrhagic Fever (CCHF) is a tick-borne disease caused by Crimean-Congo Haemorrhagic Fever virus (CCHFV), a negative sense RNA virus in the family *Nairoviridae*; order *Bunyavirales*.^1^, ^2^, ^3^, ^4^ Human CCHFV infections range in severity^5^; in many cases symptoms following infection are mild, presenting as non-specific febrile illness.^5^^,^^6^ In more severe cases, CCHFV can cause haemorrhagic, gastrointestinal and neurological symptoms, with potentially fatal outcomes.^7^, ^8^, ^9^ Mortality rates are typically estimated to range between 5 and 30% in CCHFV disease outbreaks.^10^^,^^11^

The major host of CCHFV belongs to the *Hyalomma* genus of ticks.^12^, ^13^, ^14^ CCHFV is maintained in an enzootic cycle, with infection routes of humans occurring through the bite of infected ticks or contact with the bodily fluids of infected animals or humans.^15^, ^16^, ^17^, ^18^ Of the WHO blueprint priority pathogens,^19^^,^^20^ CCHFV covers one of the vastest geographic ranges^5^^,^^21^^,^^22^ due to tick reservoirs and climate change facilitating further spread of the *Hyalomma* genus ticks.^23^^,^^24^ There remain no approved targeted treatments for CCHF, though the nucleoside analogue drug ribavirin is sometimes given despite its questioned clinical efficacy.^25^, ^26^, ^27^, ^28^ Similarly, at present there is a single vaccine against CCHFV which has limited licensure in Eastern Europe due to possible safety concerns and poor immunogenicity.^29^^,^^30^ A number of experimental vaccines have been tested in preclinical studies and demonstrated protection in animal models against CCHFV-mediated disease but progress has been slow in advancing promising candidates towards human clinical trials.^31^, ^32^, ^33^, ^34^, ^35^ As such, there is a continued need for the development of scalable, affordable and effective vaccines against CCHFV to protect susceptible individuals in endemic regions.

As demonstrated during the SARS-CoV-2 pandemic, adenoviruses are an established vaccine platform technology that can be rapidly produced at scale and have the capability to induce robust immune responses after vaccination.^36^, ^37^, ^38^, ^39^ Adenoviruses have been used extensively to develop vaccines against outbreak pathogens and include the human adenoviral vector-based Zabdeno for Ebola virus,^40^ and the ChAdOx1-vectored Oxford/AstraZeneca vaccine against SARS-CoV-2.^39^^,^^41^ The use of chimpanzee adenoviruses (ChAd) are advantageous as they can circumvent possible issues with pre-existing immunity to human adenovirus vectors.^42^, ^43^, ^44^ Though cross reactivity between simian adenoviruses exists, a previous study in humans has displayed anti-vector neutralising antibodies induced after ChAdOx2 adenoviral vector vaccination did not induce a detectable increase in titres to the related ChAdOx1 vector within individuals that had pre-existing immunity.^45^, ^46^, ^47^

Modified Vaccinia Ankara (MVA) is another viral vectored platform that has been evaluated extensively as a vaccine technology against infectious diseases such as influenza, HIV and malaria.^48^^,^^49^ The poxvirus vector can stably express large transgene inserts and elicits both humoral and cellular responses.^50^^,^^51^ However, repeat vaccination is often required with the MVA platform technology to achieve sufficient levels of immunogenicity.^52^^,^^53^ An MVA vaccine expressing the CCHFV glycoprotein precursor (GPC), encoding the structurally important Gn and Gc glycoproteins, has been shown to be both immunogenic and protective in a lethal mouse challenge model when given as a homologous prime-boost.^54^^,^^55^ The Gc and Gn virion envelope glycoproteins are key immune targets due to their likely role in mediating cell attachment and membrane fusion,^56^^,^^57^ with human survivors of CCHF often displaying long-term cellular and humoral immunity towards these antigens.^58^

Here, we evaluated the immunogenicity and protection of a replication-deficient adenoviral-vectored vaccine against CCHF, expressing the CCHFV GPC (ChAdOx2 CCHF), either alone or in combination with a previously described MVA CCHF vaccine.^54^ We demonstrated strong antibody responses as well as IFN-γ-mediated cellular immunity following immunisation with different combinations of these vaccine modalities in immunocompetent BALB/c and immuno-deficient A129 mice. Complete protection against CCHFV-mediated disease was achieved in the A129 lethal mouse model when given a single dose of ChAdOx2 CCHF or after receiving homologous or heterologous prime-boost vaccination regimens. These findings highlight the potential to use the ChAd platform as an effective approach for vaccine-mediated protection against CCHFV infection.

## Methods

### Viruses

CCHFV IbAr10200 strain was initially harvested from suckling mouse brain homogenates and subsequently passaged on SW13 cells. Stock concentration of the virus was determined by a focus-forming assay in Vero E6 cells, with the lowest lethal dose (LD100) found to be 10^2^ focus-forming units per ml (ffu/ml) in a volume of 100 μl.

### Construction of vectors

MVA CCHF vaccine was produced as previously reported at the UK Health Security Agency (Porton Down, UK).^54^ In brief, plasmids containing the full-length M segment open reading frame (ORF) nucleic acid sequence of CCHF strain IbAr10200, codon-optimised for humans, were constructed by Gateway recombination and then transfected into MVA-infected BHK-21 cells to generate a recombinant MVA virus, named here as MVA CCHF.

ChAdOx2 CCHF viral-vectored vaccine was generated at the Viral Vector Core Facility (VVCF) of the Jenner Institute, University of Oxford, UK. ChAdOx2, a replication-defective E1/E3 deleted chimpanzee adenovirus vector, was produced from the wild-type replication-competent isolate AdC68 (species adenovirus E, also known as SAdV-25 and Pan 9), with further modification to the E4 region.^44^ The nucleic acid sequence of the full length CCHFV M segment ORF (GenBank accession number U39455.2) was codon-optimised for expression in humans and inserted between the human cytomegalovirus major immediate early long promoter (IE CMV), which includes intron A and two tetracycline operator 2 sites, and the bovine growth hormone polyadenylation signal in a shuttle plasmid. The expression cassette was then inserted into the E1 locus *via* site specific recombination technology between the ChAdOx2 destination DNA BAC vector and the shuttle plasmid. The viral vector was then rescued and grown in the HEK293 derived TRex cell line (Invitrogen, Cat. R71007) prior to CsCl purification and sterile filtration.

### Animals and immunisations

**Fig. 1:**
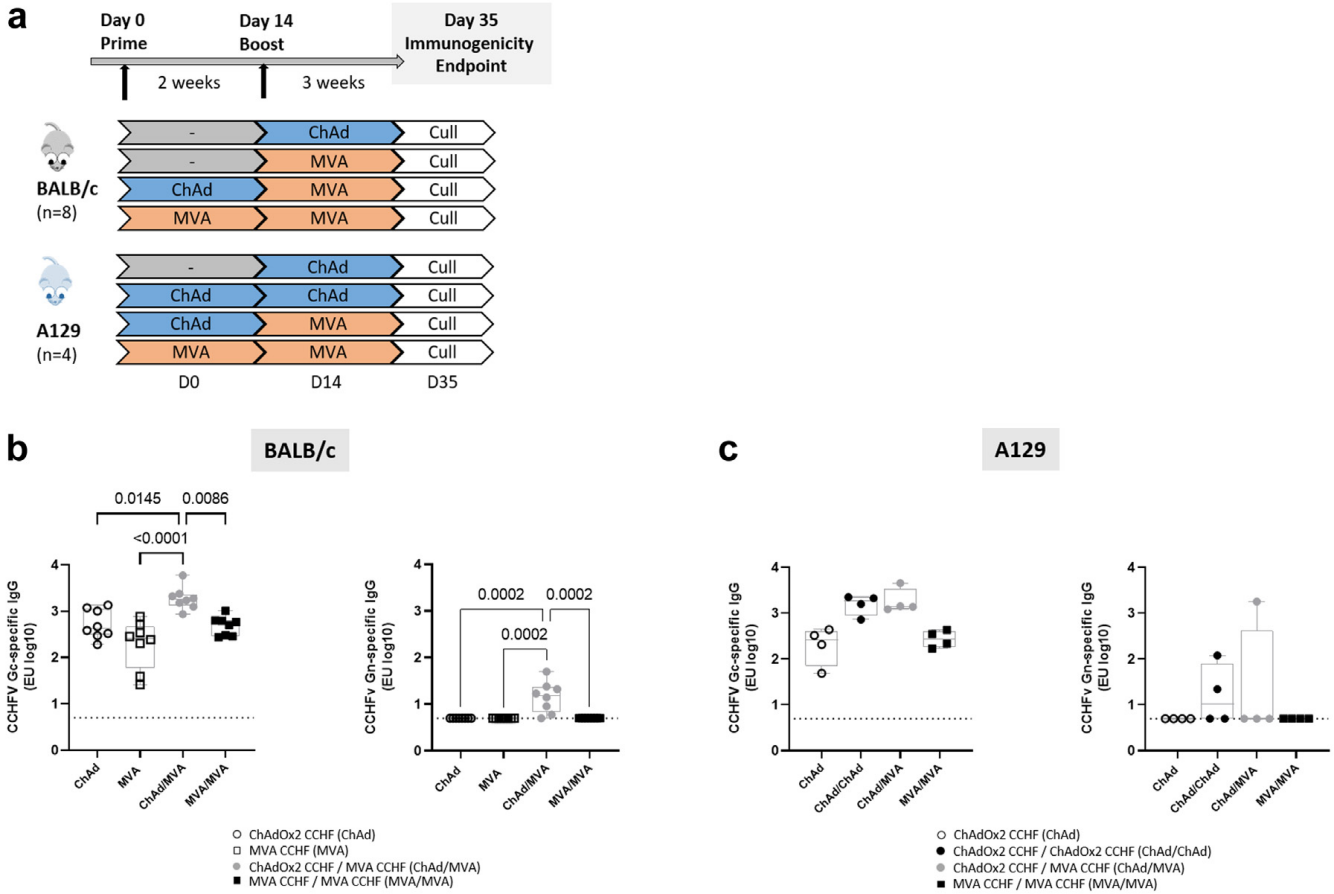
**CCHFV-specific IgG responses following ChAd and MVA immunisation.** Immunisation schedules of the two mouse strains **(a)**. Prime-boost regimens received prime vaccination on day 0 of experiment, and prime only regimens and prime-boost regimens received prime and boost vaccination respectively on day 14. Antibody responses were measured in the serum of BALB/c (n = 8) **(b)** and A129 (n = 4) mice **(c)** collected 3 weeks after the final immunization. CCHFV Gc-specific (left panel) and Gn-specific (right panel) IgG responses were quantified by standardised ELISA. Individual data points expressed as logarithmic ELISA units (EU log10) are shown here as a scatter dot plot with boxes showing the median and interquartile range and whiskers showing minimum and maximum. For (**b**, left panel) significant differences were determined by a one-way ANOVA with Tukey post-hoc analysis and data in graphs (**b**, right panel) and **(c)** analysed with Kruskal–Wallis test with Dunn's correction for multiple comparisons between vaccination groups. Dotted lines represent the quantified level of response from control immunised mice.

Female A129 (IFNα/βR^−/−^) mice and female BALB/c mice, aged 6–9 weeks and 8 weeks respectively, were obtained from Marshall BioResources (UK). All vaccines were formulated in endotoxin-free PBS and administered by intramuscular injection (IM) into the caudal aspect of the hind leg. Five groups of BALB/c mice (n = 8 per regimen) were immunised with 1 × 10^7^ plaque-forming units (PFU) MVA (MVA CCHF or MVA GFP control), or 5 × 10^7^ infectious units (IU) of ChAdOx2 (ChAdOx2 CCHF or ChAdOx2 GFP control) (Fig. 1a). 5 groups of A129 mice (n = 4 for immunogenicity and n = 6 for challenge per regimen) were vaccinated with 1 × 10^7^ PFU MVA CCHF or 5 × 10^7^ IU ChAdOx2 (ChAdOx2 CCHF or ChAdOx2 GFP control) (Fig. 1a). A total volume of 100 μl was delivered to each BALB/c mouse, with 50 μl across two hindlimb sites, and A129 mice received a total volume of 50 μl. Animals for immunogenicity testing were euthanised, and terminal bleeds and spleen harvesting carried out 3 weeks after final dose given (day 35). In prime only regimens, mice were vaccinated with ChAdOx2 or MVA, and terminal bleeds and spleen harvesting at 21 days post immunisation (d.p.i). In heterologous or homologous prime-boost regimens, mice were vaccinated with ChAdOx2 or MVA (at doses indicated) and ChAdOx2 or MVA boosted 14 days later; mice were terminally bled and spleens harvested at 21 d.p.i. (post-boost).

### IFN-γ ELISpot assay

Spleens collected from animals were homogenised and red blood cells lysed prior to splenocyte resuspension in RPMI medium (Sigma–Aldrich) supplemented with 5% FBS, 2 mM l-Glutamine, 100 U penicillin and 0.1 mg/mL streptomycin, 50 μM 2-mercaptoethanol and 25 mM HEPES solution (Sigma–Aldrich). ELISpot assays were performed using PVDF microtitre plates pre-coated with antibody AN18 (Mabtech, Cat. 3321-3-250). Splenocytes seeded at 2 × 10^6^ cells/ml were stimulated with pools of peptides spanning the CCHF glycoprotein gene at 2.5 μg/mL/peptide (Mimitopes), a positive control, or negative control. Plates were incubated for 18 h at 37 °C, 5% CO_2_ in a humidified incubator. IFN-γ spot forming cells were detected by staining membranes with anti-mouse IFNγ biotin (1 mg/mL) (R46A2, Mabtech Cat No. 3321-6-250) followed by streptavidin-alkaline phosphatase (1 mg/mL, Mabtech Cat No. 3310-8-1000) and development with AP conjugate substrate kit (BioRad). Plates were read and spots counted using an automated ELISpot scanner (Cellular Technologies Limited) and analysed using ImmunoSpot 5.0.9.21. Background values were subtracted from the responses measured in media control wells containing no peptides, and IFN-γ responses were reported as spot forming units (SFU) per 10^6^ cells.

### Standardised enzyme-linked immunosorbent assay (ELISA) for detection of CCHFV Gc or Gn-specific total IgG

CCHFV anti-Gc and anti-Gn IgG were measured by an indirect ELISA with a standard curve from a reference serum pool of mice with high CCHFV specific-IgG responses. Nunc MaxiSorp 96-well plates (ThermoFisher Scientific) were coated with CCHFV Gc protein with human Fc-tag or Gn protein with a His-tag (The Native Antigen Company, Cat No. REC31696 and REC31615) at 1 μg/mL in phosphate buffered saline (PBS) and stored overnight at 4 °C for a minimum of 16 h. Plates were washed with PBS containing 0.05% Tween20 and wells then blocked with 1% Blocker™ Casein in PBS (ThermoFisher Scientific) for 1 h at room temperature (RT). Mouse sera, individually diluted in casein to be within the linear range of the standard curve, were added to plates in duplicate. A two-fold serial dilution of the standard positive pool was added to the plates for anti-Gn detection or a three-fold dilution series to plates for anti-Gc detection to produce ten standard points that were assigned arbitrary ELISA units (EUs). Two internal controls of the positive standard serum pool and wells containing casein alone were included on each plate. Plates were incubated for 2 h at RT and then washed and alkaline phosphatase (AP)-conjugated goat anti-mouse IgG (Sigma–Aldrich, Cat No. A1418), diluted 1:5000 in casein, was added to all wells for 1 h at RT. Plates were washed and developed by addition of pNPP substrate (Sigma–Aldrich). Optical density (OD) values for each well were measured at 405 nm using a Bio-tek ELx800 Microplate Reader. OD values were fitted to a 4-Parameter logistic model (Gen5 v3.09, BioTek) standard curve. Test sera arbitrary units (EU) were calculated from their OD values using the parameters estimated from the standard curve.

### Gc-specific IgG avidity ELISA

The Gc-specific IgG antibody avidity was determined by a sodium thiocyanate (NaSCN) displacement ELISA. Plate coating and blocking were performed the same as the Gc-specific total IgG ELISA. Sera were diluted in casein to normalise titres to give a level of 1 total IgG EU. After incubation for 2 h at RT and washing, NaSCN (Sigma–Aldrich) was added in an increasing concentration gradient from 0 M to 6 M NaSCN in duplicate wells down the plate. Plates were incubated for 15 min at RT followed by washing and the secondary antibody addition and development performed the same as for the total IgG ELISA. Avidity was measured using the intercept of the OD_405_ curve for each sample with the line of 50% reduction of the OD_405_ in the 0 M NaSCN wells for each sample.

### Gc specific-IgG subclass ELISAs

For testing of IgG subclasses, mouse sera were diluted individually in casein to 1 total IgG EU. ELISA methodology was the same for IgG subclasses as per the total IgG ELISA, with the following described exceptions. The samples added to the plates were incubated for 2 h at 37 °C before respective secondary IgG subclass-specific antibodies were added, using AP-conjugated goat anti-mouse IgG1, IgG2a, and IgG2b (Southern Biotech, Cat No. 1071-04, 1081-04 and 1091-04, respectively) diluted 1:4000 in casein and 1:1000 for AP-conjugated goat anti-mouse IgG3 (Abcam, Cat No. 98705), then incubated at 37 °C for 1 h. The results of the IgG subclass ELISA are presented using OD values. All antibodies were validated by the commercial supplier.

### CCHFV TecVLP pseudotype neutralisation assay

Neutralisation capacity of the antibodies from vaccinated mice were assessed using a pseudotype neutralisation assay described by Zivcec and colleagues that utilises CCHF transcription- and entry-competent virus-like particles (tecVLPs).^59^ Briefly, tecVLPs were produced by transfection of HuH7.5 (kindly gifted by McKeating and colleagues of the Nuffield Department of Medicine, Oxford) with CCHFV helper plasmids encoding the bacteriophage T7 RNA polymerase, strain IbAr10200 GPC, NP, the RNA-dependent RNA polymerase helper plasmids, and the pL-Luc minigenome plasmids (kindly shared by Zivcec and colleagues of the NIH) using TransIT-LT1 Transfection Reagent and in the weight ratio as previously described.^60^ Transfection media was replaced with fresh DMEM with 10% FBS (DMEM-10%) after 24 h, and cell supernatants containing tecVLP were collected following an additional 48 h incubation. For the pseudovirus neutralization assay, heat-inactivated sera were individually diluted in DMEM with 5% FBS (DMEM-5%) and then mixed at a 1:1 ration with fresh undiluted tecVLP and incubated for 1 h to allow neutralisation. The serum-tecVLP mixes were then applied to A549 cells (kindly gifted by Spencer and colleagues of the Nuffield Department of Medicine, Oxford) and incubated for 24 h at 37 °C. After 24 h, the cells were washed with PBS and fresh DMEM-5% added to each well, and then incubated for a further 24 h at 37 °C. Cell lysate was removed and the cell monolayer assayed with Nano-Glo Luciferase Assay System (Promega), with luminescence intensity measured using a Varioskan Flash luminometer (ThermoFisher Scientific). Data were normalised using positive and negative control well values, and neutralising activity measured by non-linear regression to determine the half maximal inhibitory concentration (IC50) of the normalised raw data using GraphPad Prism software (version 9).

### Challenge of A129 mice with CCHFV

For the A129 mice to be challenged (n = 6 per group), a 100 μl volume of 200 ffu CCHFV was intradermally administered in the upper medial area of the back, 22 d after the final vaccine dose was given (day 36). Following challenge, all mice were weighed and rectal body temperatures recorded daily for 20 days. Mice were also observed daily for abnormal clinical signs, with increased monitoring for one-week post–challenge. Animals exhibiting moderately abnormal clinical signs, such as loss of 10% body weight, lethargy or immobility, were euthanised when they were deemed to have reached human endpoints.

### Necroscopy

Necroscopy was performed for all A129 mice euthanised post–challenge, either when reaching humane endpoints or on the scheduled end of study (day 56). Samples of blood, spleen and liver were collected and stored at −80 °C for subsequent analysis. In addition, representative samples of spleen and liver sections were fixed in 10% neutral buffered formalin (NBF) for histopathological examination.

### Viral load quantification by RT-PCR

Once thawed in 2 mL microtubes, the spleen and liver samples were weighed, 1.5 mL of PBS added to each tube and the samples manually homogenised through a 440 μM polyester mesh (Costar). 140 μL of liver, spleen, or blood was added to 560 μL of RLT buffer (Qiagen) plus beta-mercaptoethanol, mixed by inverting, and incubated at RT for ≥10 min. 560 μL of 70% ethanol was added to each tube and the samples mixed by inverting. The tubes were centrifuged briefly and 700 μL of supernatant transferred to a QiaShredder (Qiagen) and centrifuged at full speed for 2 min. The supernatant was transferred to an S-block for RNA extraction using the KingFisher Flex Purification system (ThermoFisher Scientific) and the BioSprint 96 One-For-All Vet Kit (Indical); total RNA was eluted in 50 μL nuclease-free water and stored at −80 °C until analysis. Samples were analysed by qRT-PCR using the TaqMan™ Fast Virus 1-Step Master Mix (ThermoFisher) for 45 cycles using the fast cycling mode [reaction volume 20 μL] with primers and probes targeting the S segment of CCHF^61^ and a 10-fold serial dilution of CCHF S segment synthetic RNA [1.0 × 10^6^ to 1 copy μL^−1^].

### Histological analysis

Spleen and liver samples were immersed in 10% NBF for 7 days, before being trimmed and processed to paraffin wax. Sections were cut at approximately 3–5 μm thick and stained with haematoxylin and eosin (HE) prior to being examined by light microscopy. Using light microscopy, a qualified pathologist assessed the presence and severity of CCHFV-associated lesions in the HE stained sections of liver and spleen from each animal using a subjective scoring system [normal (0), minimal (1), mild (2), moderate (3), marked (4)].

Immunohistochemistry was performed to detect viral antigen in sections of the spleen and liver samples that were formalin-fixed and paraffin-embedded. These sections were mounted on positively charged X-tra Adhesive slides (Leica Biosystems), deparaffinised and rehydrated. Immunohistochemical staining was undertaken using a BOND-MAX Immunostainer (Leica Microsystems) and a Novacastra Bond Intense R (Leica Biosystems) detection kit. A heat-induced epitope retrieval cycle with buffer ER1 R (Leica Biosystems) was performed for 10 min. Slides, including positive and negative controls, were incubated with rabbit serum (4%) (Abcam) for 20 min followed by an avidin/biotin blocking stage (15 min each) (Abcam). Polyclonal antibody raised in sheep immunised against recombinant CCHFV nucleoprotein (kindly provided by Dr John Barr, University of Leeds, UK) was incubated with the tissue for 30 min, followed by a biotinylated rabbit anti-sheep polyclonal antibody (Abcam) at a dilution of 1∶500, for 10 min. Haematoxylin was used as the counterstain. Positive and negative control slides were included. Immunolabelled slides were evaluated using light microscopy, with a scoring protocol used to evaluate the degree of viral antigen staining [occasional single cell staining (1); scattered, positive staining (2); frequent, scattered staining (3); and marked, patchy to diffuse staining throughout the tissue (4)]. The evaluations were performed with the pathologist blinded to animal and treatment details to prevent bias.

### Statistical analysis

A minimum of 4 mice per group were used in all experiments to ensure appropriate sample size. This was determined based on our previous experience and articles assessing the immunogenicity of adenoviral vectors that had displayed 4 mice per group gives 80% power to detect a two-fold change in T cell or antibody responses.^62^ Group sizes were increased to 8 for BALB/c mice to compensate for the potential loss from the development of spontaneous neoplasia which are common in BALB/c mice and unrelated to the experimental procedures. Data were analysed using GraphPad Prism version 9 (GraphPad Software Inc., California, USA). Antibody and neutralisation titres were log10 transformed prior to statistical analysis. Data were first tested for Gaussian distribution by implementing Shapiro–Wilk test, followed by determination of significant differences between vaccine regimen groups by ordinary one-way ANOVA with Tukey post-hoc analysis where data was normally distributed, or by using Kruskal Wallis test with Dunn's correction for multiple comparisons for non-parametric datasets. Correlations were analysed using Pearson test. *P* values less than 0.05 were considered statistically significant.

### Ethics statement

These studies were approved by the UK Health Security Agency, Porton Down, UK, Ethical Review Process and the Home Office, UK, *via* project licence number P82D9CB4B. Work was performed in accordance with the Animals (Scientific Procedures) Act 1986 and the Home Office (UK) Code of Practice for the Housing and Care of Animals Used in Scientific Procedures (1989). Batches of animals were given a minimum of one week to acclimatise prior to being randomly split into groups and initiation of experiments. Mice were kept in negative pressure flexible isolators under climate-controlled conditions in a Containment Level 4 facility, and food and water were freely available. All efforts were made to reduce animal suffering including minimising manipulations and endpoints limited to a moderate severity rating. All animals were humanely euthanised at the end of each experiment by inducing unconsciousness using isoflurane gaseous anaesthesia, followed by cervical dislocation.

### Role of funders

The funder was not involved in study design, data collection, data analysis, manuscript reviewing or editing of the final version.

## Results

### Heterologous prime-boost immunisation enhances CCHFV Gc-specific binding antibodies in BALB/c and A129 (IFN-α/βR^−/−^) mice

The ChAdOx2 CCHF vaccine consists of the replication-deficient simian adenovirus vector ChAdOx2 described previously.^44^^,^^45^ Similar to MVA CCHF, ChAdOx2 CCHF here was constructed to contain the full-length CCHFV M segment open reading frame sequence that encodes the glycoprotein precursor (GPC) and contains the two mature glycoproteins Gc and Gn that were the focus of the post-vaccination immunogenicity profiling in this study. Detailed immunisation schedules with heterologous, homologous and a single-dose of ChAdOx2 CCHF and MVA CCHF vaccine modalities in BALB/c mice and A129 mice are schematically depicted in Fig. 1a. For simplicity, within the results section ChAdOx2 CCHF has been abbreviated as ChAd whilst MVA CCHF has been abbreviated as MVA, as indicated in the figure symbol legend.

BALB/c mice developed humoral responses against CCHFV Gc following immunisation with either heterologous, homologous or single-dose vaccination with ChAdOx2 and MVA vaccine modalities (Fig. 1b, left panel). CCHFV Gc-specific IgG responses were highest in the heterologous prime-boost viral vector regimen (Fig. 1b, left panel); the ChAd/MVA regimen displayed a significantly increased response compared with both the single dose and homologous MVA immunisation regimens. There were no significant differences in CCHFV Gc-specific IgG responses between the ChAd or MVA prime only regimens and the homologous MVA prime-boost. The ChAd/MVA prime-boost regimen also displayed significantly higher IgG responses towards the CCHFV Gn compared to the prime only immunisation regimens and MVA/MVA that showed no CCHFV Gn-specific IgG response (Fig. 1b, right panel).

Similar to the immunocompetent mice, A129 (IFN-α/βR^−/−^) mice exhibited CCHFV Gc-specific humoral responses following immunisation with all the ChAdOx2 and MVA vaccine modalities (Fig. 1c, left panel). A homologous regime of ChAd/ChAd, as well as a heterologous regimen of ChAd/MVA, induced higher levels of IgG against CCHFV Gc compared to those induced by a ChAd prime only and MVA/MVA regime in A129 mice (Fig. 1c, left panel). However, the variations in Gc-specific total IgG response between groups in A129 mice were not significantly different. Low or undetectable levels of CCHFV Gn-specific IgG response were induced by the different immunisation regimens in A129 mice, with no significant differences between the groups (Fig. 1c, right panel).

### ChAdOx2 CCHF immunisation induces high-avidity antibodies with neutralisation capacity in BALB/c and A129 mice

**Fig. 2:**
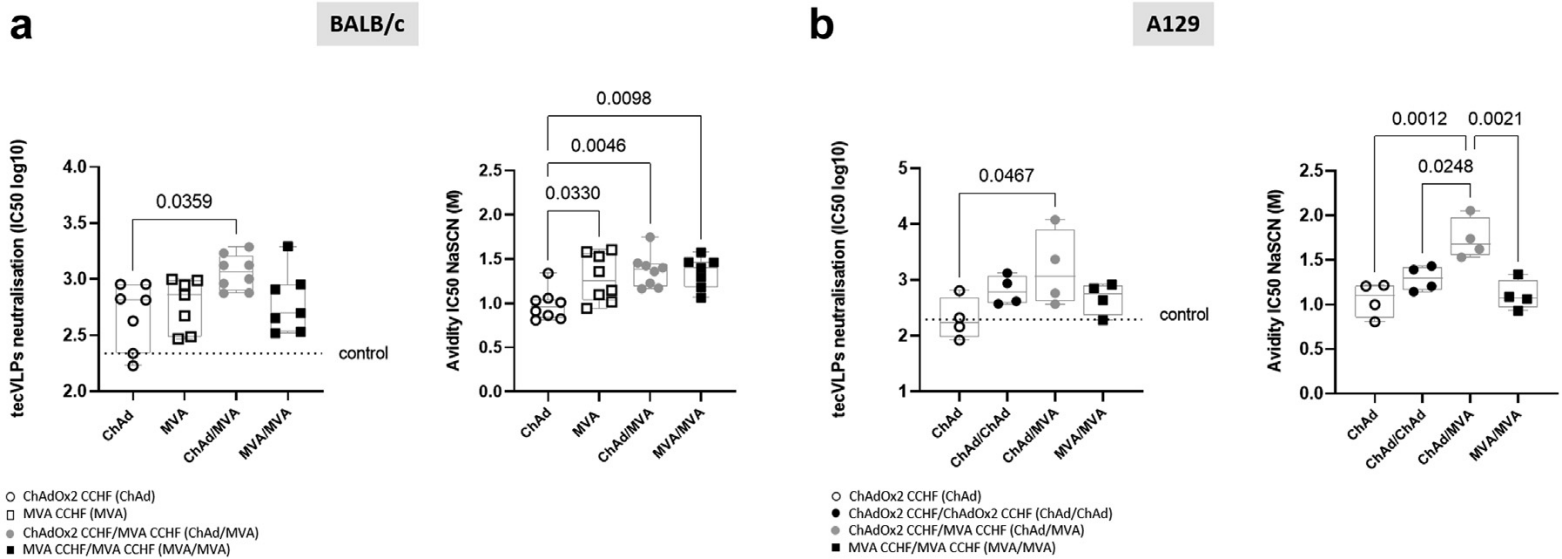
**Measurement of CCHFV-specific antibody-mediated neutralisation and avidity.** Antibody neutralisation responses and avidity were measured in the serum of **(a)** BALB/c (n = 8) and **(b)** A129 (n = 4) mice collected 3 weeks after the final immunisation. Neutralisation was assessed by measuring inhibition of CCHFV tecVLPs entry into A549 cells, shown by individual data points expressed as logarithmic IC50 values (left panels). Avidity of CCHFV Gc specific IgG responses was measured using a NaSCN chemical displacement ELISA (right panels). Individual data points are shown here as a scatter dot plot with boxes showing the median and interquartile range and whiskers showing minimum and maximum. Significant differences were determined by one-way ANOVA with Tukey post-hoc analysis. Dotted lines represent the quantified level of response from control immunised mice.

**Fig. S1 correl:**
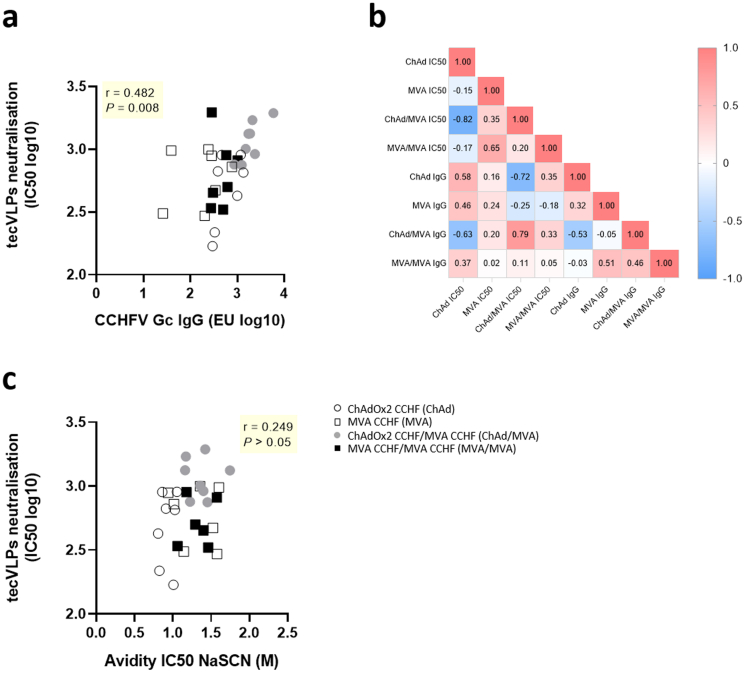
**Figure S1. Detailed serology following immunisation of BALB/c mice with various ChAd and MVA regimens**. **(a** and **b)** Relationship between levels of CCHFV-Gc specific IgG and tecVLP neutralisation IC50 correlation displayed by scattered dot plot (left panel) and correlation heatmap matrix (right panel). **(c)** Relationship between levels of tecVLP neutralisation IC50 and avidity IC50, displayed as scattered dot plot. Statistical analyses were performed using two-tailed Pearson correlation test.

Antibody neutralization capacity was evaluated by a pseudotype neutralisation assay that generates CCHF transcription- and entry-competent virus-like particles (tecVLP); these have been shown to be morphologically similar to live CCHFV by possessing the structural proteins.^59^^,^^60^ In both BALB/c (Fig. 2a, left panel) and A129 mice (Fig. 2b, left panel), ChAd/MVA prime-boost immunisation showed significantly greater neutralisation of tecVLPs entry compared to the ChAd prime only group. In BALB/c mice the neutralising response was correlated with IgG antibody levels (r = 0.482 and *P* < 0.008, Pearson test) (Fig. S1a). Comparing between the BALB/c immunisation groups, the highest correlation between IgG antibody levels and neutralisation was noted in animals immunised with ChAd/MVA (r = 0.787 and *P* = 0.020, Pearson test) (Fig. S1b).

Antibody avidity was measured using a NaSCN-displacement ELISA that provides a measure of the overall strength of the polyclonal antigen-specific IgG response and can be attributed with possible enhanced antibody functionality. Avidity towards CCHFV Gc in BALB/c mice was broadly similar across all prime-boosted groups, with significant differences seen for groups of mice immunised with ChAd/MVA, MVA/MVA and MVA prime only compared to ChAd prime only (Fig. 2a, right panel). There was no correlation between avidity and neutralisation in BALB/c mice (Pearson r = 0.249, *P* > 0.05) (Fig. S1c). In A129 mice, the greatest IgG avidity was seen in the heterologous ChAd/MVA regimen, which showed significantly greater avidity than any other experimental group tested (Fig. 2b, right panel). Avidity of Gn-specific antibodies was not measured in either mouse strain due to low level of response for total IgG towards Gn.

### A mixed profile of IgG subclasses are induced by ChAdOx2 CCHF immunisation

**Fig. 3:**
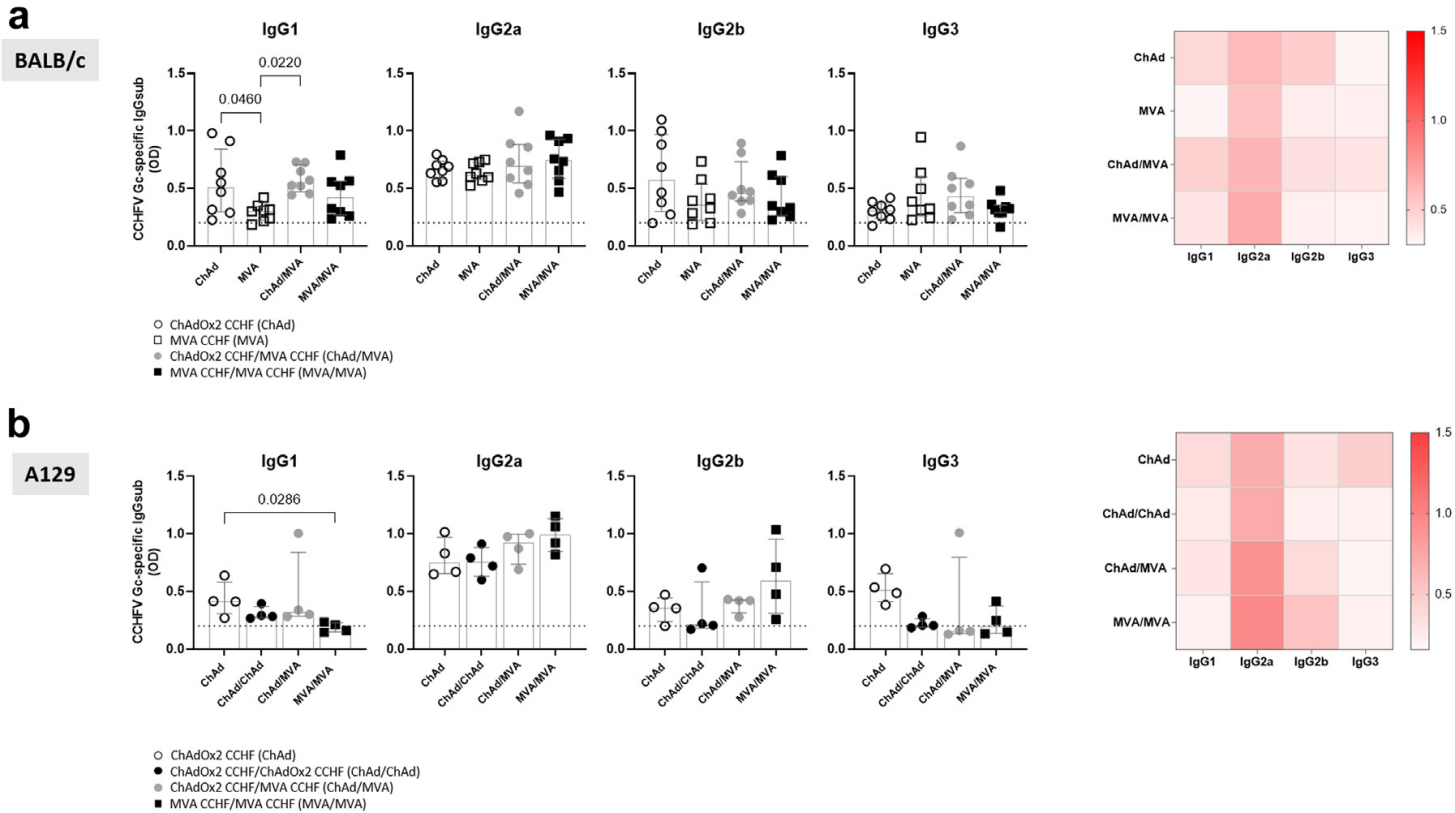
**Detection of IgG subclasses in BALB/c and A129 mice immunised with ChAd and MVA regimens.** Samples with detectable CCHFV Gc-specific responses were normalised and diluted to 1 EU. IgG subclasses were quantified by optical density and data displayed as scattered dot plots with bars showing the median and IQR, and as heatmap with median OD values of each group. Individual data points represent OD of a single mouse. BALB/c mice data (n = 8) **(a)** in each graph were analysed with a one-way ANOVA with Tukey post-hoc analysis, and A129 (n = 4) **(b)** data analysed with Kruskal–Wallis test followed by a post hoc Dunn's multiple comparison test to compare differences between vaccination groups. Dotted lines represent the assay limit of quantification.

Further characterisation of the CCHFV Gc-specific IgG response revealed a mixed profile of IgG subclasses in both mouse strains, with mainly IgG2a being induced in all animals across the immunisation regimens (Fig. 3a and b). The IgG1 response in BALB/c mice was higher in groups primed with ChAd, with significantly increased responses in ChAd/MVA and ChAd prime only regimens compared to the MVA only regimen (Fig. 3a). By contrast, IgG1 remained low in A129 mice amongst immunisation groups with the only difference seen for the significantly higher IgG1 response in the ChAd prime compared to MVA/MVA (Fig. 3b).

### ChAdOx2 CCHF immunisation elicits IFN-γ cellular responses predominantly towards CCHFV GPC

**Fig. 4:**
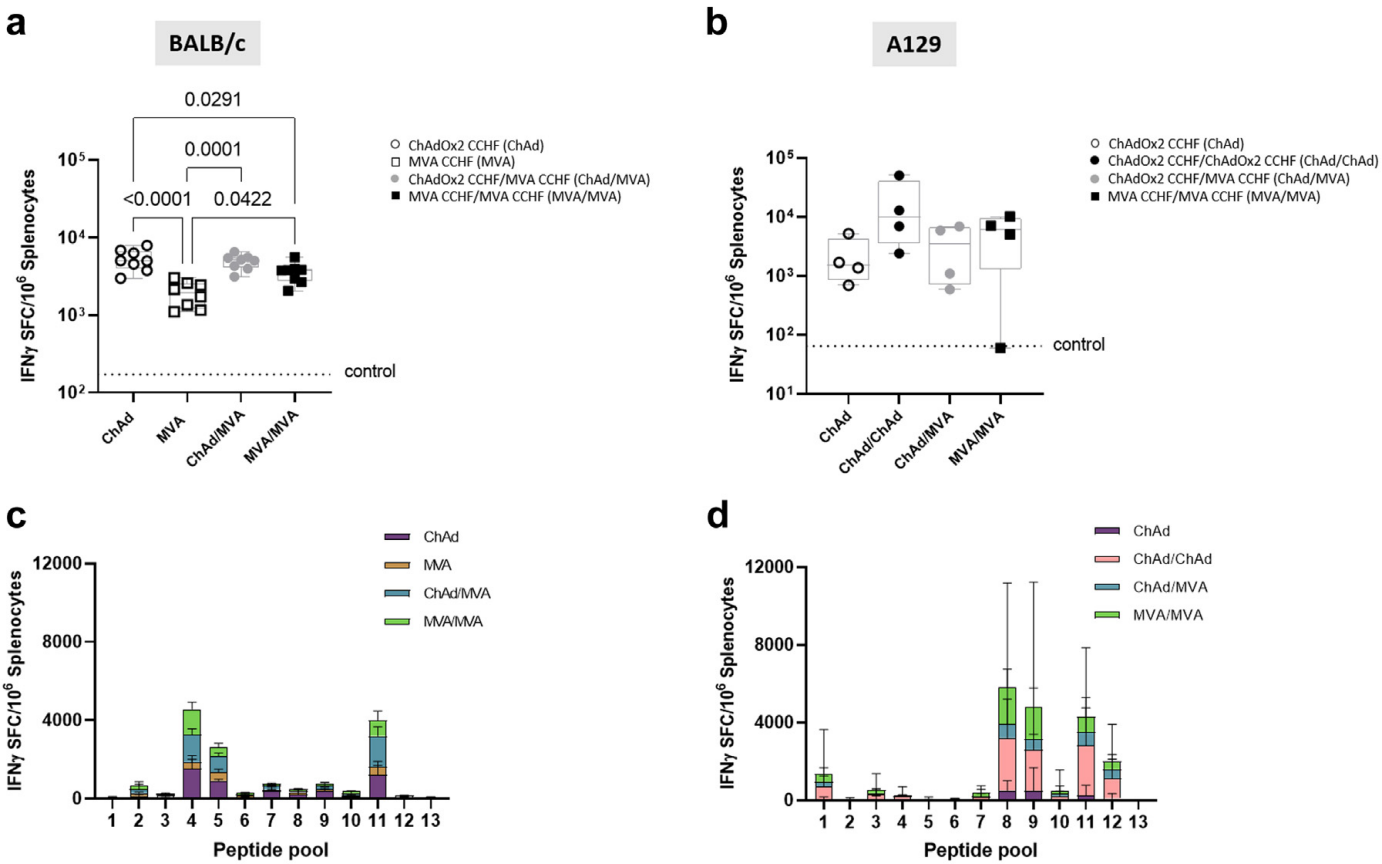
**CCHFV-specific cellular responses in mice immunised with ChAd and MVA regimens.** CCHFV antigen-specific IFN-γ responses in mouse splenocytes were assayed by IFN-γ ELISPOT assays. The summed IFN-γ ELISPOT responses in BALB/c **(a)** and A129 mice **(b)** are displayed as individual data points as a scatter dot plot with boxes showing the median and interquartile range and whiskers showing minimum and maximum. Responses to individual peptide pools are displayed by stacked bars of IFN-γ responses in BALB/c **(c)** and A129 mice **(d)**, with lines showing the median with IQR. Significant differences were determined by one-way ANOVA with Tukey post-hoc analysis. Dotted lines represent the quantified level of response from control immunised mice.

In both mouse strains, all of the vaccine regimens induced CCHFV-specific cellular responses that were measured by IFN-γ ELISPOT to investigate T-cell-mediated immunity (Fig. 4a and b). In BALB/c mice, a single dose immunisation of ChAd was significantly better in inducing IFN-γ than a single administration MVA prime regime and MVA/MVA prime-boost (Fig. 4a). The single dose ChAd response was also equivalent to the cellular responses induced by ChAd/MVA regimen (Fig. 4a). IFN-γ ELISPOT responses in BALB/c mice were greatest in the experimental peptide pools 4, 5 and 11 corresponding to the C-terminus of GP38, N-terminus of Gn and to the central region of Gc, respectively (Fig. 4c).

The highest measured IFN-γ response in A129 mice was observed in the group receiving a homologous ChAd/ChAd regime, however, there were no statistically significant differences between groups (Fig. 4b). The absence of significant difference for the variations observed in antibody and IFN-γ responses in A129 mice between the ChAd and MVA regimens assessed was likely due to either the small sample size or immunocompromised nature of the A129 mice. In A129 mice, the IFN-γ ELISPOT responses were greatest against peptide pools 8, 9, 11, and 12, which correspond to the N-terminus and central regions of CCHFV Gc (Fig. 4d).

### A129 mice immunised with ChAdOx2 CCHF are fully protected against CCHFV challenge

**Fig. 5:**
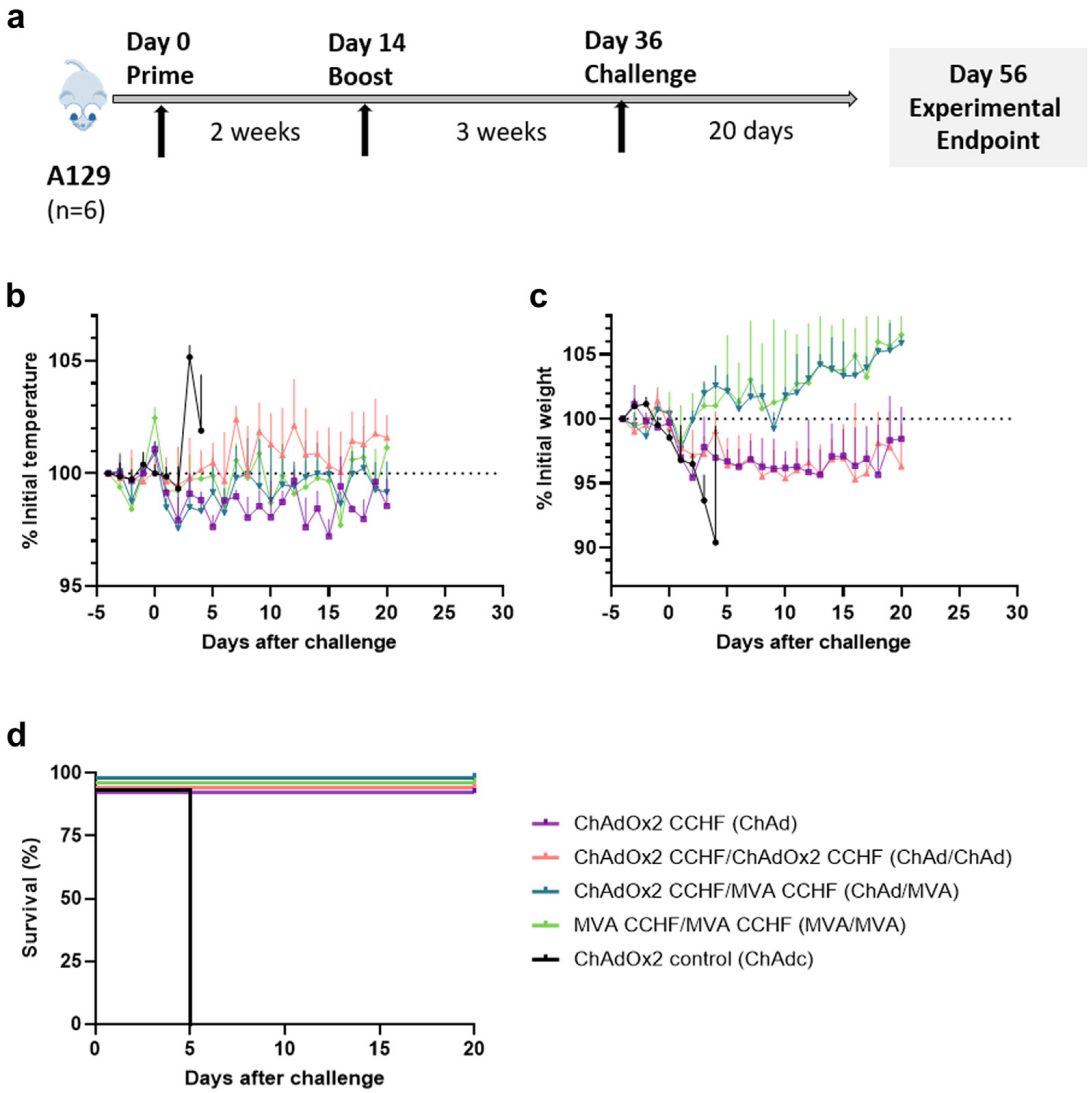
**Assessing the protective effect of ChAd and MVA regimens against challenge with CCHFV in A129 mice.** Challenge timeline overview **(a)** for A129 mice (n = 6 mice per group). Mice in prime-boost regimens received prime vaccination on day 0 of experiment. Prime only regimens and prime-boost regimens received prime and boost vaccination respectively on day 14. Mice were challenged on day 36 with a 100 μl volume of 200 ffu CCHFV that was intradermally administered. All surviving mice were euthanised on day 56. Following challenge, all mice were monitored for changes in rectal temperature **(b)** and bodyweight **(c)** that are displayed as the recorded median of each regimen group with error bars representing IQR, as well as Kaplan–Meier survival plot **(d)** displaying percentage survival up to 20 days post challenge.

A129 mice were challenged with a lethal dose of CCHFV IbAr10200 strain, 22 days post final immunisation as displayed in Fig. 5a. Mice immunised with an unrelated ChAd control vaccine showed clinical signs of disease and by day 5 post challenge were deemed to have met humane endpoints and were euthanised. Following challenge, an elevated rectal body temperature was noted in all control mice during days 4 and 5 (Fig. 5b), and a prominent decrease in body weight up to day 5 (Fig. 5c). Conversely, mice immunised with ChAd or MVA against CCHFV had rectal temperatures within normal limits (Fig. 5b) and body weights recovered or stabilised after an initial small decrease post–challenge (Fig. 5c). As shown in Fig. 5d, all mice that received immunisation against CCHFV survived until the endpoint of the experiment at 20 days post challenge (Fig. 5d).

**Fig. 6:**
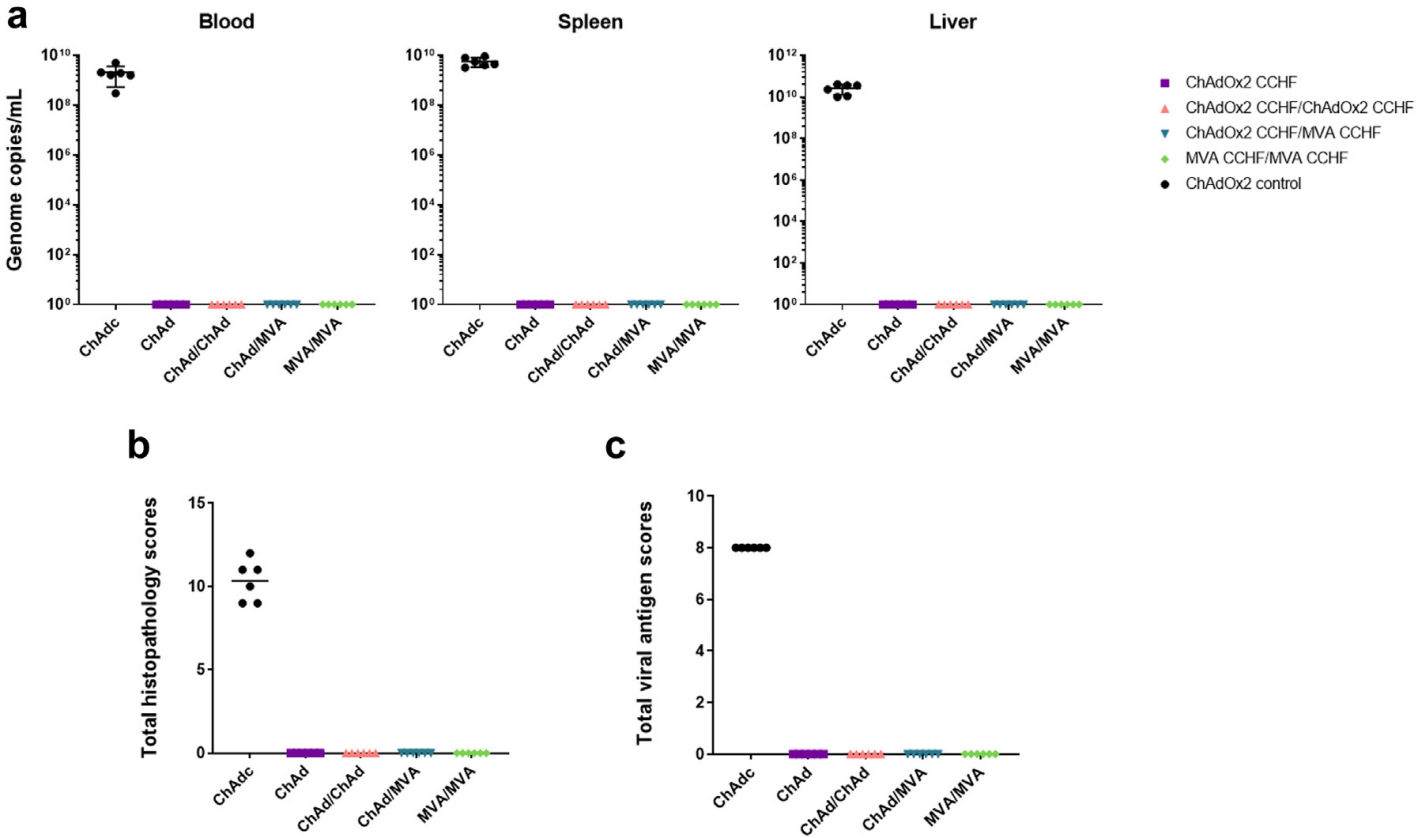
**Viral load and histopathological analysis following lethal CCHFV challenge of A129 mice immunised with ChAd or MVA.** A129 mice (n = 6 per group) vaccinated with ChAd or MVA CCHF were challenged with CCHFV, and twenty days post–challenge (day 56) all surviving animals were killed humanely and tissue samples taken for analysis. **(a)** Graphs show RNA levels measured in blood, spleen and liver by RT-PCR analysis for CCHFV gene expression, with control vaccinated mice analysed for viral RNA 5 days post challenge after reaching humane endpoints. Each point represents the mean value of triplicate measurements in an individual animal. Lines show mean ± standard deviation. Sections of spleen and liver were fixed, stained, and examined by pathology; graphs show combined subjective scores of all animals in all groups for **(b)** microscopic changes from histopathology on the liver and spleen using a scoring system [normal (0), minimal (1), mild (2), moderate (3), marked (4)], and **(c)** total score for viral antigen staining of the liver and spleen, with the system [occasional single cell staining (1); scattered, positive staining (2); frequent, scattered staining (3); and marked, patchy to diffuse staining throughout the tissue (4)]. Horizontal bars represent median value.

Samples of blood, spleen and liver were collected from euthanised animals at the end of the study (20 days post–challenge), although samples from control mice were taken on day 5 post–challenge after euthanasia was performed as a result of reaching humane endpoint criteria. The levels of CCHFV RNA were compared by RT-PCR and demonstrated that in the blood, spleen and liver there was an absence of viral RNA in all mice immunised against CCHFV, unlike ChAd control immunised mice that displayed high viral RNA in blood, spleen and liver (Fig. 6a).

**Fig. 7:**
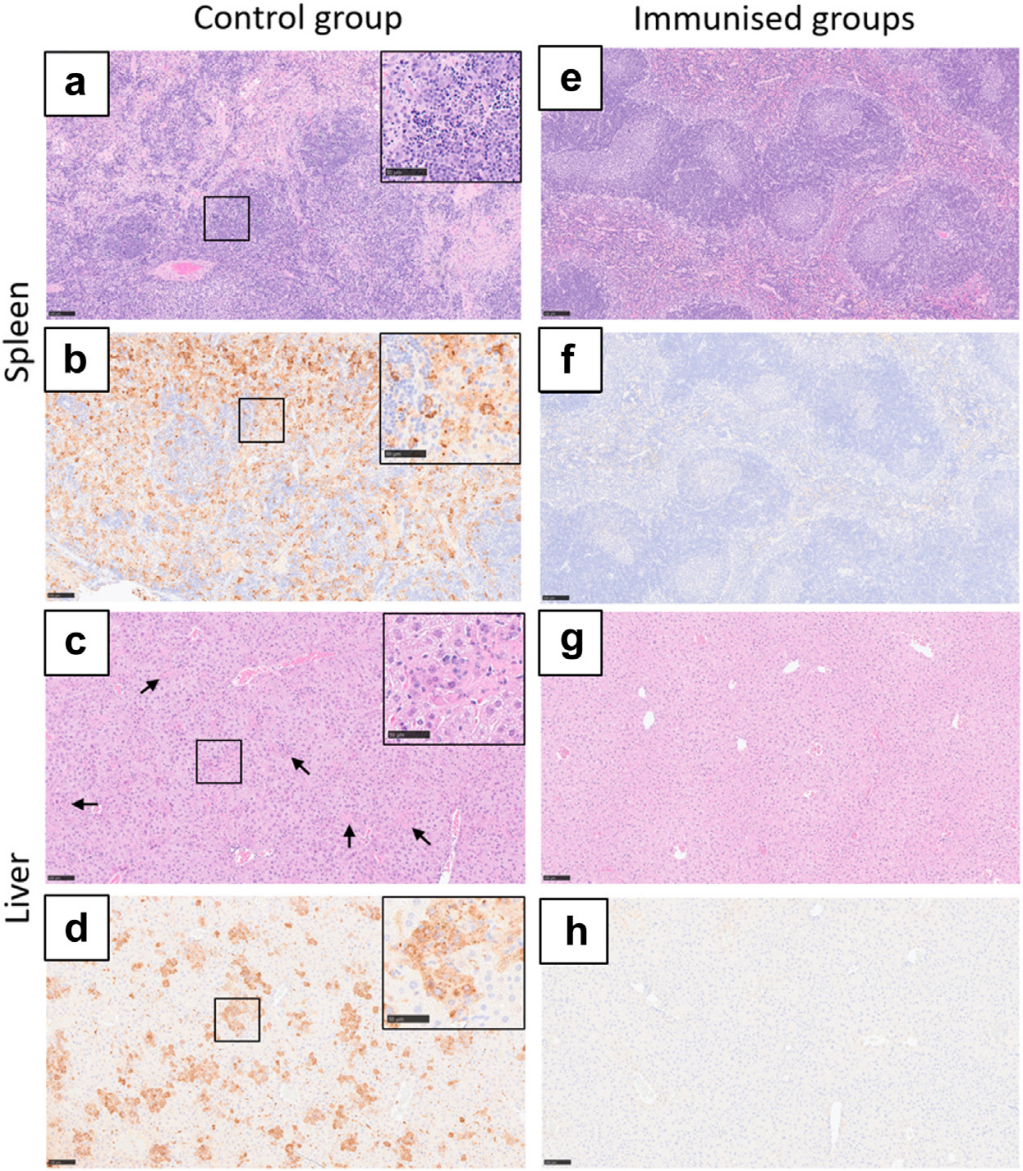
**Representative images of the histopathological changes in the spleen and liver of A129 mice after challenge with CCHFV.** A129 mice were challenged with a lethal dose of CCHFV 21 days after prime or prime-boost immunisation with ChAd or MVA. Haematoxylin and eosin-stained sections and IHC- stained sections for viral antigen were examined from mice that were euthanised after meeting either the study or humane endpoints. In the spleen of the control mice **(a)**, there was mild to marked lymphocytolysis in the white pulp, with prominent, tingeable body macrophages; in the red pulp, a variable increase in macrophages and some neutrophils, were noted. Inset, higher power image showing lymphocyte apoptosis and tingeable body macrophages in the white pulp. HE. Prominent, diffuse, staining of viral antigen was noted within the parenchyma **(b)**, most prominent in cells in the red pulp. Inset, higher power image of stained cells. IHC. In the liver of the control mice **(c)**, there was a moderate to marked, multifocal hepatocyte necrosis scattered randomly throughout the parenchyma (arrows) and accompanied by variable numbers of inflammatory cells, primarily neutrophils. Inset, higher power image of necrotic hepatocytes. HE. Prominent, diffuse, staining of viral antigen **(d)** was noted within the parenchyma, most prominent in intra-lesional hepatocytes. Inset, higher power image of cells staining positive for viral antigen. IHC. In the spleen **(e, f)** and liver **(g, h)** of all mice in the treated groups, microscopic lesions associated with CCHF infection and staining for viral antigen, were absent. HE. IHC. Scale bars are shown at 100 μM for large low power images, and 50 μM scale bars for magnified square higher power images.

Histopathological lesions consistent with CCHFV infection and positive staining of CCHFV viral antigen were observed in all animals in the ChAd control group (Fig. 6b and c, respectively, and Supplementary Table S1). Splenic lesions attributed to CCHFV infection in ChAd control immunised mice included prominent follicular lymphocyte depletion in the white pulp, comprising lymphocytolysis with karyorrhectic debris and concomitant tingeable body macrophages; lymphocytolysis, and a variable, diffuse infiltration of macrophages and neutrophils, were noted in the red pulp (Fig. 7a). The spleen of all control immunised mice displayed strong staining for viral antigen in cells throughout the parenchyma, with a predominance of staining of cells in the red pulp (Fig. 7b). In the liver of ChAd control immunised mice, microscopic changes compromised of small, multiple foci of hepatocyte necrosis and loss, affecting single or small groups of hepatocytes randomly located within the parenchyma, and characterised by cytoplasmic hyper-eosinophilia, pyknosis and loss of nuclear detail (Fig. 7c); these changes were accompanied by a variable, inflammatory cell infiltrate, primarily neutrophilic, with scattered macrophages. Strong staining of cells, indicating the presence of viral antigen, was prominent in the liver of these control immunised mice, comprising mainly intralesional hepatocytes (Fig. 7d). By contrast, CCHF-associated microscopic lesions and presence of viral antigen were absent in both the spleen (Fig. 7e and f, respectively) and liver (Fig. 7g and h, respectively) of all animals immunised with candidate vaccines expressing the CCHFV GPC.

## Discussion

There exists widespread epidemic potential for CCHFV outbreaks from the geographical expansion of CCHFV through the introduction of ticks into new regions and the continued lack of preventative or therapeutic countermeasures.^63^^,^^64^ In the current study, we report that: 1) Both anti-CCHFV humoral and cellular immunity was elicited by the ChAdOx2 CCHF vaccine in BALB/c and A129 mice after homologous prime-boost and single-dose immunisation as well as after heterologous prime-boost immunisation in combination with the MVA CCHF vaccine and; 2) ChAdOx2 CCHF protects against CCHFV-mediated disease in A129 mice when administered as a heterologous, homologous and single-dose vaccination. Present results strongly support further ChAdOx2 vaccine development against CCHF.

Prime-boost regimens are an established immunisation strategy to achieve increased potency of vaccine-induced humoral and cellular responses and more durable immune responses.^65^^,^^66^ This has included using heterologous prime-boost, with either viral vector (ChAd or MVA) for first dose and the alternative vector for second dose (e.g. MVA or ChAd, respectively).^67^, ^68^, ^69^, ^70^ We demonstrate here that all immunisation regimens elicited antibody responses, where heterologous regimens induced the highest CCHFV antigen-specific antibody responses with increased avidity and neutralising titres.

Following natural infection with CCHFV, low antibody responses were shown to correlate with more severe outcomes of the disease,^71^, ^72^, ^73^, ^74^ whilst various studies have indicated different levels of importance of antibodies in controlling CCHFV infection and survival in mouse models, including a contentious role of neutralising antibodies for protection.^34^^,^^55^^,^^75^^,^^76^ After natural infection in humans, low levels of neutralising antibodies have been reported during convalescence^58^^,^^77^ and vaccination of a non-human primate model with a DNA-based CCHFV vaccine, containing plasmids encoding the GPC and NP, provided protection against CCHF and induced high antibody titres but with no neutralising activity.^35^ It was previously published that homologous prime-boosting with the MVA CCHFV GPC vaccine induced protection and comprehensive immunogenicity, although neutralising antibodies were not measured.^54^^,^^55^ Here, our findings indicate that neutralising antibodies could be important for CCHFV protection induced by ChAd or MVA vectored vaccines, though there was complete survival of A129 mice that lacked high neutralising antibodies, such as those immunised with ChAdOx2 CCHF prime-only or homologous prime-boosting. It is also possible that due to low assay sensitivity any small increases in levels of neutralising antibodies were not detected in these mice. It could be speculated that non-neutralising antibody-mediated mechanisms as well as other facets of the adaptive immune response may be sufficient for protection in this animal model. As the different vaccine regimens tested in A129 mice were all completely protective, it is not possible to deduce correlates of protection. Future dose down studies that compare the immune response in surviving mice and non-survivors will be necessary to decipher the correlates of protection for ChAdOx2 CCHF vaccines.

Both viral-vectored vaccines elicited IFN-γ cellular immune responses in both mouse strains. The predominantly IgG2a subclass profile seen here suggests a Th1 response may be induced, consistent with previous studies that have reported adenovirus and MVA viral vectors frequently display an immunological profile biased towards Th1 responses.^70^^,^^78^^,^^79^ The role and contribution of T cells in resolving infection following natural infection in humans remains unclear. Yet, human CCHF survivors have displayed memory CD8+ T cells that are detectable several years following infection,^80^ and both CD4+ and CD8+ T cells were shown to be important for survival of acute CCHFV infection in a immunocompromised mouse model.^81^

The majority of the IFN-γ responses were mapped to the CCHFV Gc in both BALB/c and A129 mice, although these cellular responses were also stimulated by the GP38 non-structural protein and Gn in BALB/c mice. The CCHFV GPC has represented an attractive target for CCHFV vaccine development as neutralising antibodies are typically generated against the Gc portion of the GPC,^31^^,^^34^^,^^82^ as well as Gc being theorised to be responsible for cell tropism by binding an unknown human cell receptor to facilitate viral entry.^83^^,^^84^ Our cellular and humoral response findings imply the Gc to be the more favourable GPC antigen for ChAdOx2 due to the increased immunogenicity compared to Gn. In spite of this, heterodimerisation of Gn with Gc is important for bunyavirus GPC protein folding and localisation.^85^^,^^86^ For instance, both CCHFV Gn and GP38 have been suggested to interact with Gc to enable viral particle assembly and infectivity.^87^^,^^88^ Thus, there may be a potential role of Gn for ensuring the GPC antigen is correctly processed and formed in CCHFV vaccines.

Significantly, immunocompromised A129 mice were protected from CCHFV lethal challenge after a single dose of ChAdOx2 CCHF and had similar levels of IFN-γ responses detected after a single immunisation with ChAdOx2 CCHF compared to prime-boost regimens. If reproducible in humans, this protective efficacy after a single dose CCHFV vaccine regimen would be beneficial for achieving population immunity more quickly and easily. An effective CCHF vaccine would ideally be used to protect high-risk populations in endemic regions and also to control CCHFV during outbreaks, in doing so mitigating the risk of severe CCHFV infections and mortality upon exposure.^5^^,^^64^ Endemic regions can often be remote and lack infrastructure; a single-dose vaccine would be more feasible for rollout to suppress these outbreaks.^89^ Many CCHF vaccine candidates, including MVA and DNA vaccines, have used prime-boost immunisation regimens to achieve full protection in mice.^35^^,^^54^^,^^90^^,^^91^ Recently, separate rVSV and VRP-based CCHF vaccines have shown complete protection in mice with a single immunisation.^31^^,^^92^ The ChAdOx2 CCHF vaccine further demonstrates that pre-clinical CCHF vaccines can confer protection with single-dose regimens and is also consistent with other emerging pathogen ChAd-vectored vaccines that were protective after one dose in animal models.^93^, ^94^, ^95^, ^96^, ^97^ In addition, ChAdOx2 as a viral vector has also been shown to be well tolerated in humans.^45^ Viral load assessment and histopathological examination results provided further evidence that the ChAdOx2 CCHF vaccine protected the vaccinated animals against overt pathology as a result of CCHFV challenge. No virus was detected at 20 days post–challenge in any of the protected animals, however we cannot preclude that there may have been some detectable virus at earlier timepoints which was then robustly cleared in animals vaccinated with candidate vaccines encoding the CCHFV GPC.

Overall, the data displayed here demonstrates that vaccination with a ChAdOx2 viral vectored vaccine targeting CCHFV induces strong antibody responses as well as IFN-γ-mediated cellular immunity. Most significantly, complete protection against CCHFV-mediated disease was achieved in the A129 lethal mouse model when they received either one or two doses of ChAdOx2 CCHF. Collectively, the findings described here demonstrate that further pre-clinical and clinical development of the ChAdOx2 CCHF vaccine candidate is warranted.

## Contributors

SD, SCG, and TL designed the study. SD, VAG, EK, KT, and RH conducted the animal procedures, sample processing and/or cellular response experimentation. ER interpreted the pathological findings. JES, CG, SM, MU, AJS, and SBR performed humoral immunogenicity experiments. JES, CG, and SBR analysed and verified the underlying data. JES, CG, SBR, and TL contributed to the writing of the manuscript; JES, SBR, and TL were responsible for the decision to submit the manuscript. All authors read and approved the final version of the manuscript submitted.

## Data sharing statement

The data supporting the findings of this study are found within the article and the supplementary material. All relevant raw data will be made available from the corresponding author upon reasonable request.

## Declaration of interests

SCG is co-founder and board member of Vaccitech and named as an inventor on a patent covering use of ChAdOx2-vectored vaccines. TL was consultant to Vaccitech. The remaining authors declare no competing interests.
