# Supplementary material for: Adenoviral vectored vaccination protects against Crimean-Congo Haemorrhagic Fever disease in a lethal challenge model
Source: eBioMedicine. 2023 Mar 17;90:104523. doi: 10.1016/j.ebiom.2023.104523 (PMC10025009; doi:10.1016/j.ebiom.2023.104523)
Supplement: Supplementary Table S1 [file mmc1.docx]

|  | | | Group | | | | |
| --- | --- | --- | --- | --- | --- | --- | --- |
| Tissue | **Description** | **Severity** | **ChAdc (day 5)** | **ChAd** | **ChAd/ChAd** | **ChAd/MVA** | **MVA/MVA** |
| Spleen | Macrophages in red and white pulp | Normal | 0 | 6 | 6 | 6 | 6 |
|  |  | Minimal | 0 | 0 | 0 | 0 | 0 |
|  |  | Moderate | 4 | 0 | 0 | 0 | 0 |
|  |  | Marked | 2 | 0 | 0 | 0 | 0 |
| Spleen | Lymphocyte apoptosis/necrosis | Normal | 0 | 6 | 6 | 6 | 6 |
|  |  | Minimal | 1 | 0 | 0 | 0 | 0 |
|  |  | Moderate | 3 | 0 | 0 | 0 | 0 |
|  |  | Marked | 2 | 0 | 0 | 0 | 0 |
| Liver | Focal hepatocyte necrosis | Normal | 0 | 6 | 6 | 6 | 6 |
|  |  | Minimal | 0 | 0 | 0 | 0 | 0 |
|  |  | Moderate | 1 | 0 | 0 | 0 | 0 |
|  |  | Marked | 5 | 0 | 0 | 0 | 0 |

**Supplementary Table 1. Severity scores for spleen and liver histopathological lesions in A129 mice post challenge.** A129 mice (n=6 per group) were vaccinated and then challenged with CCHFV 21 days post final immunisation. Mice were euthanised at 56 days post-vaccination, except ChAd control mice that reached humane endpoint and were removed prior to scheduled study end. Sections of spleen and liver were fixed, hematoxylin and eosin (HE) stained, and examined for pathology. Number of mice per group possessing severity rating are indicted.
